# Vaccination with SARS-CoV-2 variants of concern protects mice from challenge with wild-type virus

**DOI:** 10.1371/journal.pbio.3001384

**Published:** 2021-12-16

**Authors:** Fatima Amanat, Shirin Strohmeier, Philip S. Meade, Nicholas Dambrauskas, Barbara Mühlemann, Derek J. Smith, Vladimir Vigdorovich, D. Noah Sather, Lynda Coughlan, Florian Krammer

**Affiliations:** 1 Department of Microbiology, Icahn School of Medicine at Mount Sinai, New York, New York, United States of America; 2 Graduate School of Biomedical Sciences, Icahn School of Medicine at Mount Sinai, New York, New York, United States of America; 3 Center for Global Infectious Disease Research, Seattle Children’s Research Institute, Seattle, Washington, United States of America; 4 Institute of Virology, Charité-Universitätsmedizin Berlin, corporate member of Freie Universität Berlin, Humboldt-Universität zu Berlin, and Berlin Institute of Health, Berlin, Germany; 5 German Centre for Infection Research (DZIF), partner site Charité, Berlin, Germany; 6 Centre for Pathogen Evolution, Department of Zoology, University of Cambridge, Cambridge, United Kingdom; 7 Department of Pediatrics, University of Washington, Seattle, Washington, United States of America; 8 University of Maryland School of Medicine, Department of Microbiology and Immunology, Baltimore, Maryland, United States of America; 9 University of Maryland School of Medicine, Center for Vaccine Development and Global Health (CVD), Baltimore, Maryland, United States of America; 10 Department of Pathology, Icahn School of Medicine at Mount Sinai, New York, New York, United States of America; Weatherall Institute of Molecular Medicine, UNITED KINGDOM

## Abstract

Vaccines against Severe Acute Respiratory Syndrome Coronavirus 2 (SARS-CoV-2) have been highly efficient in protecting against Coronavirus Disease 2019 (COVID-19). However, the emergence of viral variants that are more transmissible and, in some cases, escape from neutralizing antibody responses has raised concerns. Here, we evaluated recombinant protein spike antigens derived from wild-type SARS-CoV-2 and from variants B.1.1.7, B.1.351, and P.1 for their immunogenicity and protective effect in vivo against challenge with wild-type SARS-CoV-2 in the mouse model. All proteins induced high neutralizing antibodies against the respective viruses but also induced high cross-neutralizing antibody responses. The decline in neutralizing titers between variants was moderate, with B.1.1.7-vaccinated animals having a maximum fold reduction of 4.8 against B.1.351 virus. P.1 induced the most cross-reactive antibody responses but was also the least immunogenic in terms of homologous neutralization titers. However, all antigens protected from challenge with wild-type SARS-CoV-2 in a mouse model.

## Abstract 2

This study explores the immune response induced by wild type and variant SARS-CoV-2 spike proteins, and the protection that these immune responses provide against challenge with wild type virus in the mouse model.

## Introduction

Severe Acute Respiratory Syndrome Coronavirus 2 (SARS-CoV-2) emerged in late 2019 in Wuhan, China. Since then, the virus has caused the Coronavirus Disease 2019 (COVID-19) pandemic leading to approximately 5 million official deaths globally (as of November 2021). While coronaviruses usually mutate more slowly than other RNA viruses due to the proof-reading activity of their replication machinery [1], viral variants started to emerge in the summer of 2020 in humans and mink in Europe [2–4]. In late 2020, additional variants, termed variants of concern (VoCs) emerged in the United Kingdom [5], in South Africa [6], and in Brazil [7]. These variants, B.1.1.7, B.1.351, and P.1, are more infectious than wild-type SARS-CoV-2 and feature extensive changes in both the receptor binding domain (RBD) and the N-terminal domain (NTD) of the spike protein. These 2 domains harbor the vast majority of neutralizing epitopes [8–14], and, consequently, it has been observed that—especially for B.1.351—the neutralizing activity of wild-type postinfection and postvaccination sera is reduced [15–18]. In addition, efficacy and effectiveness of vaccines against B.1.351 have been shown to be somewhat reduced, depending on the type of vaccine platform used [19,20]. For one currently licensed vaccine, the efficacy against B.1.351 was lost [21]. Updated vaccines based on variant spike sequences are currently being tested by vaccine producers and may be licensed in the future for variants that escape vaccine-induced immunity to an even larger degree, e.g. the B.1.1.529 (Omicron) variant. However, the process of updating vaccine antigens to match circulating variants is not as straightforward as it seems. Several variants might circulate simultaneously, making it difficult to choose the right antigen for optimal protection. Of course, multivalent vaccines that include more than one variant antigen can be formulated, but this increases complexity and decreases the amount of vaccine doses that can be manufactured. Understanding the antigenic relationship between variants is therefore of high importance.

Here, we vaccinated mice with recombinant spike proteins from the wild-type Wuhan-1 strain, B.1.1.7, B.1.351, and P.1 and assessed the resulting cross-neutralization in the sera. Furthermore, we challenged the animals with a wild-type strain (SARS-CoV-2/human/USA/USA-WA1/2020 (WA1)) of SARS-CoV-2 to determine if variant vaccine antigens would still protect from the prototypic virus. Adjuvanted, recombinant spike proteins were chosen as antigen since they reflect vaccines currently in clinical development by Novavax, Sanofi Pasteur, and other vaccine manufacturers.

## Results

### Variant spike proteins induce cross-neutralizing antibodies in the mouse model

**Fig 1:**
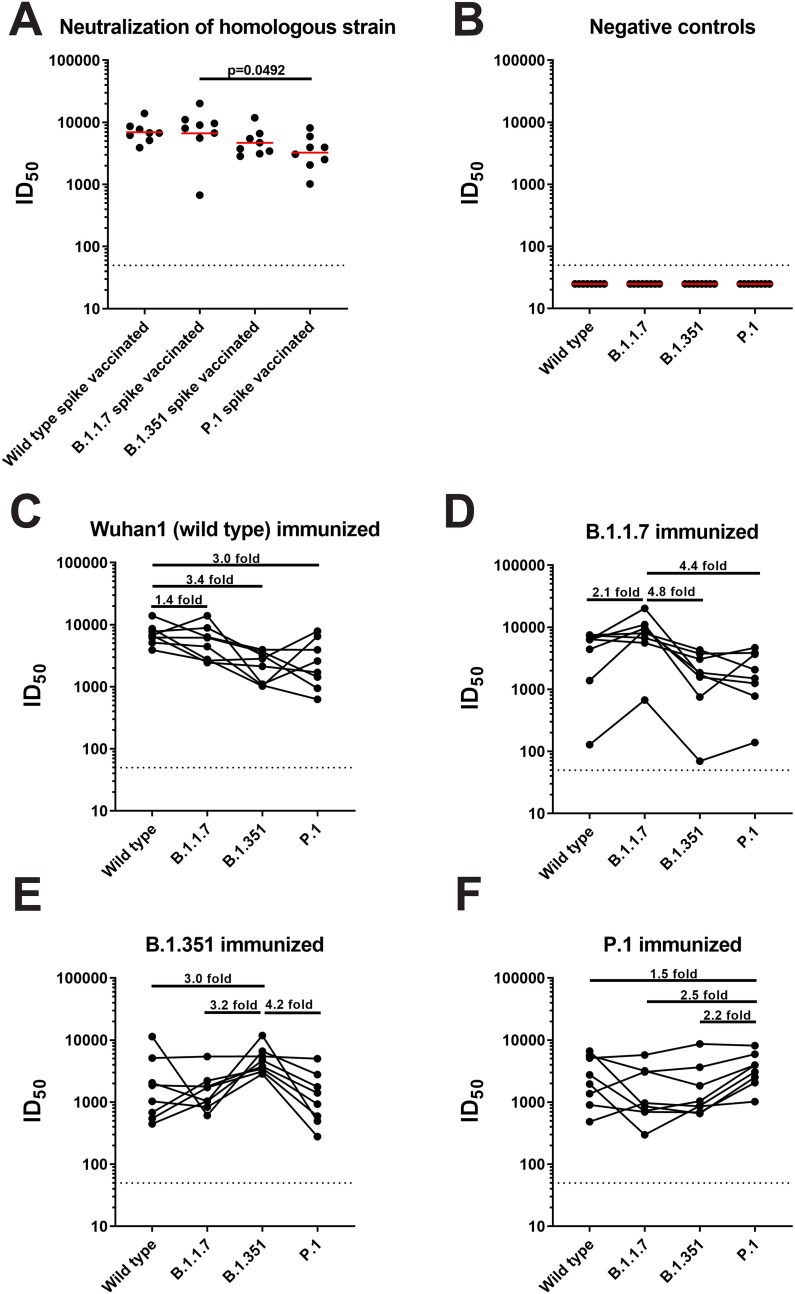
All groups of mice vaccinated with variant spike proteins can cross-neutralize wild type, B.1.1.7, B.1.351, and P.1 isolates of SARS-CoV-2. **(A)** All samples were run in a microneutralization assay with authentic SARS-CoV-2, and neutralization activity of each group against the homologous strain is shown. **(B)** Serum from the negative control group was also tested against isolates of wild-type SARS-CoV-2, B.1.1.7, B.1.351, and P.1, and the ID50s are shown. **(C-F)** Sera from mice vaccinated with wild-type spike protein **(C)**, B.1.17 spike protein **(D)**, B.1.351 spike protein **(E)**, and P.1 spike protein **(F)** were tested against wild type, B.1.1.7, B.1.351, and P.1 isolates of SARS-CoV-2, and the calculated ID50s from neutralization curves are depicted in each graph. The dashed line on each graph indicated the LOD. The differences in neutralization of different variant viruses are indicated by horizontal lines, and the fold differences in neutralization are also shown. Statistical significance was tested with an ANOVA corrected for multiple comparisons. *P* values are shown for comparisons that resulted in statistical significance. Underlying raw data can be found in the S1 Data. LOD, limit of detection; SARS-CoV-2, Severe Acute Respiratory Syndrome Coronavirus 2.

First, we vaccinated BALB/c mice twice with adjuvanted recombinant spike proteins of wild-type SARS-CoV-2 (Wuhan-1), B.1.1.7, B.1.351, and P.1. Three weeks postboost, the animals were bled and the neutralizing activity of their serum was assessed in a well-established microneutralization assay with authentic SARS-CoV-2 [22]. When tested against the respective virus from which the vaccine antigen was derived, all animals mounted strong neutralizing antibody responses (**Fig 1A;** geometric mean neutralization titers of 6,924, 6,673, 4,690, and 3,246 for wild type, B.1.1.7, B.1.351, and P.1, respectively), while negative controls showed no neutralizing activity (**Fig 1B**). The negative control group received an irrelevant control protein, influenza virus hemagglutinin. However, there was a trend toward B.1.1.7-vaccinated animals showing higher neutralizing capacity against homologous virus as compared to the other spike variant antigens. P.1 seemed to induce the lowest neutralizing activity against homologous viruses. These differences were small and only significant for B.1.1.7 versus P.1.

As expected, when testing for cross-reactivity, the different spike proteins induced the highest neutralization titers against the homologous viruses. Sera from wild-type spike-vaccinated animals neutralized WA1 best, followed by B.1.1.7, P.1, and B.1.351 (**Fig 1C;** geometric mean neutralization titers of 6,924, 4,994, 2,071, and 2,291 for wild type, B.1.1.7, B.1.351, and P.1, respectively). Sera from B.1.1.7 vaccinated animals neutralized B.1.1.7 best, followed by wild type, P.1, and B.1.351 (**Fig 1D;** geometric mean neutralization titers of 3,207, 6,673, 1,381, and 1,518 for wild type, B.1.1.7, B.1.351, and P.1, respectively). For B.1.351-vaccinated animals, we detected the highest titers against B.1.351 followed by wild type, B.1.1.7, and P.1 (**Fig 1E;** geometric mean neutralization titers of 1,580, 1,458, 4,690, and 1,131 for wild type, B.1.1.7, B.1.351, and P.1, respectively). P.1 induced a surprisingly uniform level of immunity with the lowest drop to wild-type virus followed by B.1.351 and B.1.1.7 (**Fig 1F;** geometric mean neutralization titers of 2,235, 1,276, 1,460, and 3,246 for wild type, B.1.1.7, B.1.351, and P.1, respectively). The steepest drops in neutralization were detected for B.1.1.7 to B.1.351 (4.8-fold), from B.1.1.7 to P.1 (4.4-fold), and from B.1.351 to P.1 (4.2-fold). Importantly, we did not observe complete loss in neutralizing activity against any of the viruses.

**Fig 2:**
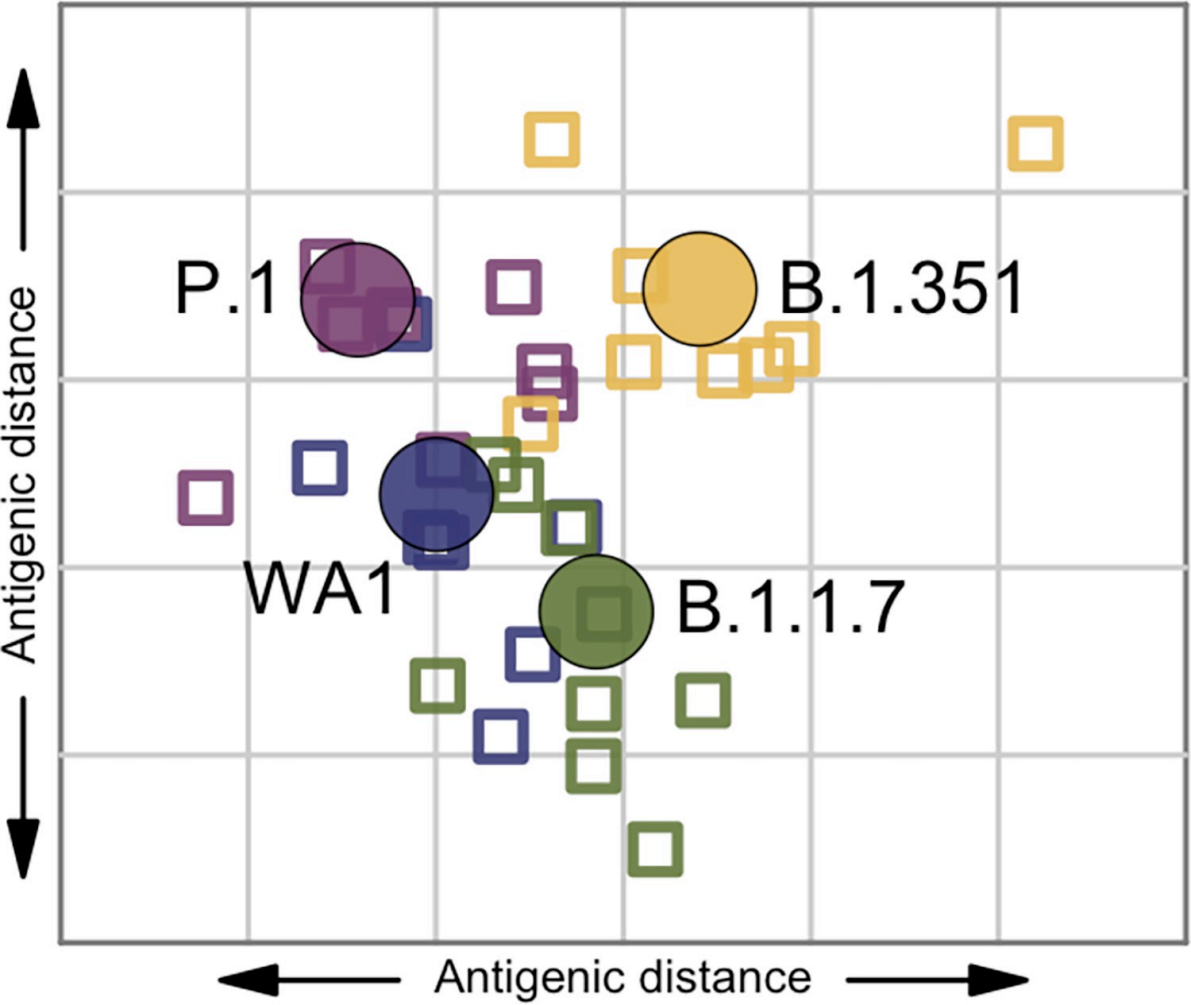
Antigenic map of WA1, B.1.1.7, P.1, and B.1.351 antigens and 31 sera. Antigens are shown as circles (WA1: blue; B.1.1.7: green; P.1: purple; B.1.351: yellow), sera as squares, in the color of the antigen they were raised against. The X and Y axes both correspond to antigenic distance, with one grid line corresponding to a 2-fold serum dilution in the neutralization assay. The antigens and sera are arranged on the map such that the distances between them best represent the distances measured in the neutralization assay. Underlying raw data can be found in the S1 Data.

We used antigenic cartography [23] to visualize the antigenic relationships between the tested viruses and sera (**Fig 2**). The B.1.351 virus is positioned furthest from the WA1 virus, and P.1 and B.1.1.7 are approximately equal distance from WA1 in opposite directions. The sera loosely cluster in the vicinity of the antigen they were raised against.

### Antibody binding is less affected than neutralization

**Fig 3:**
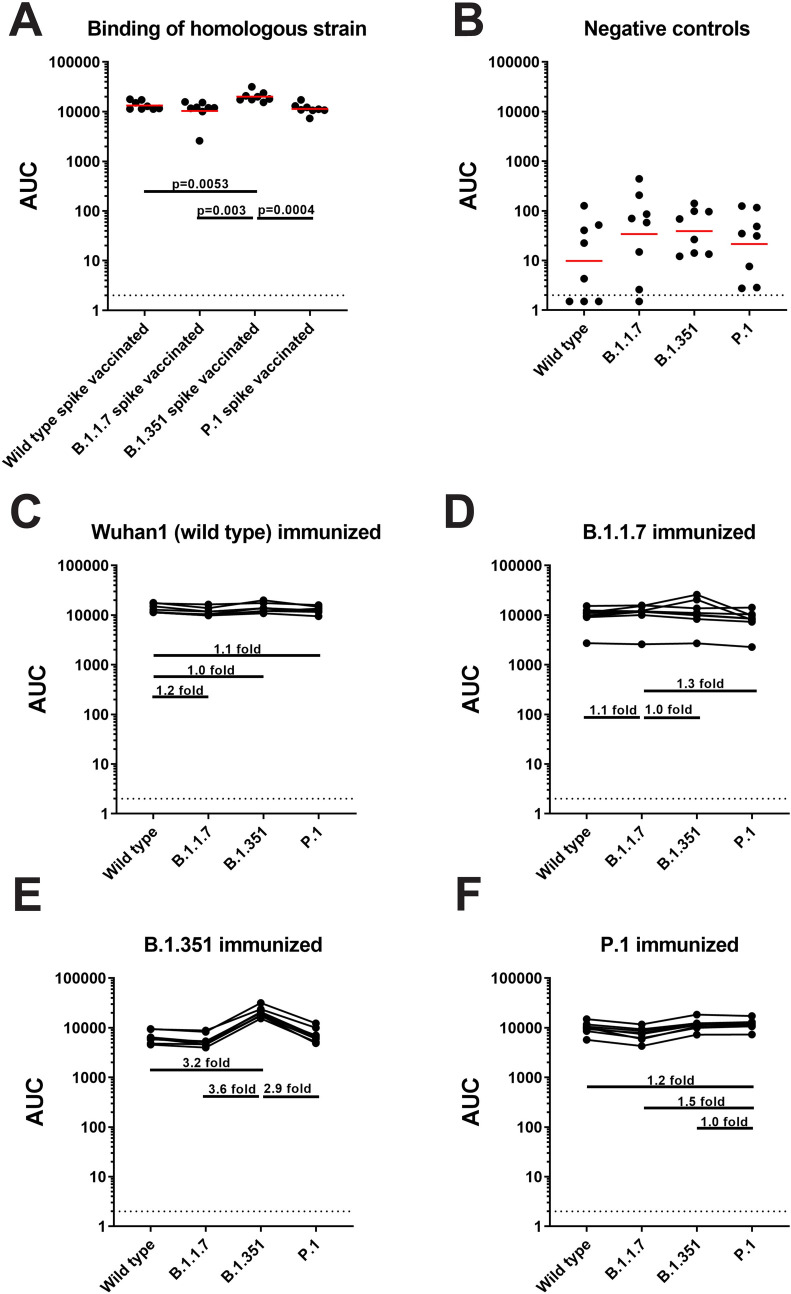
All vaccinated groups have cross-reactive antibodies in their sera against spike proteins of wild type, B.1.1.7, B.1.351, and P.1. **(A)** An ELISA was performed using sera from each group and tested for binding with the homologous spike protein, and the binding of each group against the respective spike protein is represented as AUC. **(B)** Binding of the samples in the negative control group was also tested against the spike proteins of wild-type SARS-CoV-2, B.1.1.7, B.1.351, and P.1 isolates. **(C-F)** Sera from mice vaccinated with wild-type spike protein **(C)**, B.1.1.7 spike protein **(D)**, B.1.351 spike protein **(E)**, and P.1 spike protein **(F)** were tested against the spike proteins of wild type, B.1.1.7, B.1.351, and P.1. Binding is shown as AUC, and the differences in binding are indicated by horizontal bars with the calculated fold increase or decrease. Statistical significance was tested with an ANOVA corrected for multiple comparisons. *P* values are shown for comparisons that resulted in statistical significance. Underlying raw data can be found in the S1 Data. AUC, area under the curve; ELISA, enzyme-linked immunosorbent assay; SARS-CoV-2, Severe Acute Respiratory Syndrome Coronavirus 2.

We repeated our analysis using an enzyme-linked immunosorbent assay (ELISA) with the respective spike proteins as substrates. While neutralization requires binding of antibodies to a limited number of epitopes mostly on RBD and NTD, many more binding epitopes exist on the spike protein [8]. Therefore, more even reactivity was expected. We did detect differences in reactivity when binding was tested against the respective matched spikes (**Fig 3A**; geometric mean area under the curve (AUC) values of 13,328, 10,317, 20,086, and 11,373 for wild type, B.1.1.7, B.1.351, and P.1, respectively), but while these differences were statistically significant in 3 cases, they were relatively small. However, it seemed that vaccination with B.1.351 induced slightly more homologous binding antibodies compared to the other immunogens. Low background reactivity was detected in sera of the control animals (**Fig 3B**).

Both wild-type spike and B.1.1.7 spike induced relatively even binding antibody responses (**Fig 3C and 3D**; wild type: geometric mean AUCs of 13,328, 11,545, 13,942, and 12,513 for wild type, B.1.1.7, B.1.351, and P.1, respectively; B.1.1.7: geometric mean AUCs of 9,237, 10,317, 10,765, and 7,807 for wild type, B.1.1.7, B.1.351, and P.1, respectively) with a maximum fold reduction of 1.2- and 1.3-fold, respectively. A stronger reduction was detected when B.1.351 was used as immunogen with 3.2-fold and 3.8-fold reduction in binding to wild-type and B.1.1.7 spike, respectively (**Fig 3E**; geometric mean AUCs of 6,352, 5,535, 20,086, and 6,990 for wild type, B.1.1.7, B.1.351, and P.1, respectively). The drop for P.1 was smaller (2.9-fold). P.1 also induced comparable binding antibody response with a maximum fold reduction of 1.5-fold against B.1.1.7 (**Fig 3F**; geometric mean AUCs of 9,811, 7,377, 11,437, and 11,373 for wild type, B.1.1.7, B.1.351, and P.1, respectively).

These discrepancies between neutralization and binding antibody profiles allowed us to calculate ratios between binding and neutralizing antibodies. The best (higher) ratios (indicating a higher proportion of neutralizing antibodies) were found in sera from wild-type and B.1.1.7-vaccinated mice (**S1 Fig**). For each vaccination group, the ratio was always best against the homologous virus and dropped with antigenic distance (**S1 Fig**). The most stable ratio was observed for P.1-vaccinated animals (**S1 Fig**).

### All spike-vaccinated animals are protected against challenge with wild-type SARS-CoV-2

**Fig 4:**
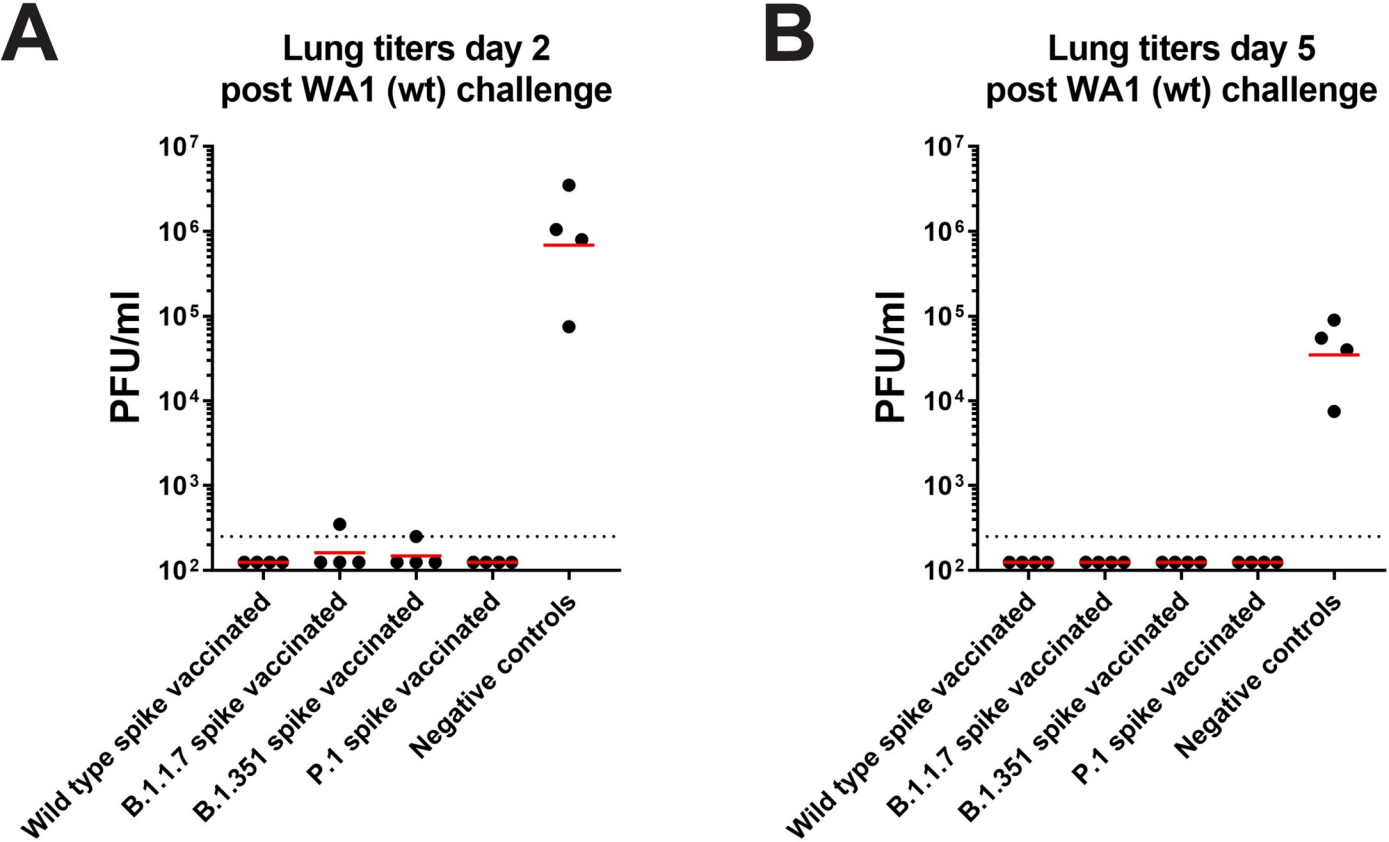
Vaccination with spike proteins of B.1.1.7, B.1.351, and P.1 protects against challenge with wild-type SARS-CoV-2 in a mouse model. **(A, B)** All groups of vaccinated mice were challenged with authentic SARS-CoV-2 after sensitization with AdV-hACE2 five days prior to infection. After infection, viral loads in the lungs were quantified via a plaque assay on day 2 **(A)** and day 5 **(B)**. Underlying raw data can be found in the S1 Data. AdV-hACE2, adenovirus-human angiotensin converting enzyme 2; PFU, plaque-forming unit; SARS-CoV-2, Severe Acute Respiratory Syndrome Coronavirus 2; wt, wild type.

Finally, we wanted to assess if the induced neutralizing antibody responses can protect animals from challenge with prototypic SARS-CoV-2 strain WA1. Since BALB/c mice are not susceptible to this virus, they had to be presensitized via intranasal transduction with adenovirus expressing human angiotensin converting enzyme 2 (hACE2) before challenge, as previously described. The main readout for the challenge experiment were virus titers in the lungs of infected animals. On day 2 postchallenge, control animals showed high viral loads in their lungs (approximately 10^6^ plaque forming units per ml of lung homogenate) (**Fig 4A**). In contrast, no virus was detected in wild-type and P.1 spike-vaccinated animals. For B.1.1.7 and B.1.351 spike-vaccinated animals, one animal per group showed traces of virus replication in the lung, but titers were barely above the limit of detection. On day 5 postinfection, no virus was detectable in the lungs of vaccinated individuals, while control animals still showed high virus loads (**Fig 4B**).

## Discussion

The emergence of SARS-CoV-2 variants is concerning both in terms of infection control (because many variants are more infectious), as well as in terms of vaccine effectiveness, due to the potential for immune escape. While several vaccines, which are either in clinical development or already in use, show good efficacy or effectiveness against most variants [19,20,24,25], the vaccine antigens may need to be updated at some point to cover variants that evade neutralizing antibodies more efficiently, like B.1.1.529 (Omicron). However, if variants cocirculate, it will be difficult to select the “right” variant that induces good immune responses across the board. Here, we have tested the cross-neutralization activity of wild type, B.1.1.7, B.1.351, and P.1 adjuvanted protein vaccines in the mouse model. We found that P.1 induces the most balanced immune response across the four tested antigens, supported by four sera raised against P.1 positioned centrally in the antigenic map between WA1, B.1.351, and P.1. Interestingly, while B.1.351 and P.1 share 2 of their RBD mutations and have a mutated residue at position 417 in common (although to different amino acids), a sharp drop in neutralizing activity from B.1.351 to P.1 was observed. However, this relationship was asymmetric, since the drop from P.1 to B.1.351 was much smaller. When considering the results from this study, P.1 should likely be the chosen immunogen for updated vaccines in our mouse model. Our mouse data are similar compared to human cross-neutralization data with the same four variants published by Liu and colleagues [26]. This suggests that mice may be a good model system to study antigenic variability among variants. Such model systems are of importance, as they ensure the continued availability of first-infection sera for the characterization of novel variants. However, there may be subtle differences between mouse strains and certainly between mice and humans, which need to be further explored. Interestingly, binding was much less affected than neutralization. There could be several reasons for that. One reason could be reduced antibody affinity that still allows binding but does not support neutralization anymore. Another reason could be that only a small number of epitopes are targeted by neutralizing antibodies while many more epitopes exist (especially outside the RBD and NTD) that are bound by nonneutralizing antibodies. If some of the neutralizing antibodies lose binding, the effect on overall binding is small because so many nonneutralizing antibodies still bind different epitopes on the very large spike. Similar findings have recently been reported in humans [27]. Importantly, all spike antigens, independently of the lineage, provided robust protection against challenge with the prototypic WA1 strains, suggesting that “updated” vaccines—especially if they induce high neutralizing antibody titers—would sufficiently protect against most other circulating variants as well as the prototypic SARS-CoV-2 strain. Of note, we do not think that the low virus titer (barely above the limit of detection) in one of the B.1.1.7 and in one of the B.1.351-vaccinated animals changes this conclusion. While challenge with variant viruses was not possible at this point in time in the selected mouse system (some variants bind to mouse ACE2 also, making comparisons complicated), we plan to evaluate cross-protection in more detail in the hamster model in the future. However, our work addresses the very pertinent question if a variant-based vaccine (as being developed by several vaccine producers) would protect against a mismatched/heterologous virus.

Our work here has focused on neutralizing and binding antibodies, which have been implicated as correlates of protection for SARS-CoV-2 vaccine-induced immunity [28,29], and reduction in neutralizing antibodies in sera from convalescent individuals and vaccinees against variants has been observed. However, T-cell responses very likely contribute to protection from COVID-19 as well. We have not analyzed T-cell responses in our experimental animals, but others have shown that the impact of variants on these responses is minimal [30]. Another caveat of our study is that we were not able to include B.1.617.2, B.1.617.1, C.37, and B.1.1.529 in our analysis even though these are currently important variants.

In summary, we found that neutralizing titers are always highest against the homologous virus but that antigenic relationships are not necessarily symmetric and that some variant spike proteins induced more balanced responses (e.g., P.1) than others (B.1.351 and B.1.1.7). In addition, the drop in binding antibody is much lower than the drop in neutralizing activity. Nonneutralizing binding antibodies have been shown to play an important role in protection for other diseases caused by virus infections including Ebola virus disease and influenza A and B viruses [31–34]. The maintenance of binding antibody and T-cell responses against variants could partially explain the maintenance of vaccine effectiveness, despite the occasional steep drops in neutralizing antibody titers.

## Materials and methods

### Recombinant proteins

All recombinant proteins were expressed and purified using Expi293F cells (Life Technologies, Carlsbad, CA), as described in detail previously [22,35]. The spike gene of each respective variant (EPI_ISL_703454, EPI_ISL_745160) was cloned into the pCAGGS vector and used to transfect cells. The cleavage site was deleted by removing the arginine residues, and prolines were added to position 986 and 987 to stabilize the spike trimer. The supernatant was clarified on day 4 posttransfection via centrifugation at 4,000*g* for 20 minutes. Ni^2+^-nitrilotriacetic acid (NTA) agarose (Qiagen, Hilden, Germany) was used to purify the protein, as described before [36,37]. EPI_ISL_792680 was cloned into pcDNA3.4 for transient transfection. The endogenous leader peptide was replaced with the tPA secretion signal, 8XHIS and AviTag epitopes were appended, and the substitutions noted above introduced. The spike trimer was expressed by transient transfection in 293F cells and purified by affinity chromatography as previously described [38].

The proteins used for ELISA were purchased from Sino Biological and include the following: 40589-V08B6, 40589-V08B7, 40589-V08B8, and 40589-V08B1.

### Cells and viruses

Vero.E6 cells (ATCC CRL-1586, clone E6) were kept in culture using Dulbecco’s modified Eagle medium (Gibco, Waltham, MA), which was supplemented with 10 mL of Antibiotic-Antimycotic (100 U/ml penicillin–100 μg/ml streptomycin–0.25 μg/ml amphotericin B; Gibco), 10% of fetal bovine serum (FBS; Corning, Corning, NY), and 1% HEPES (N-2-hydroxyethylpiperazine-N-2-ethane sulfonic acid; Gibco). Wild-type SARS-CoV-2 (isolate USA-WA1/2020), hCoV-19/South Africa/KRISP-K005325/2020 (B.1.351, BEI Resources NR-54009), hCoV-19/Japan/TY7–503/2021 (P.1, BEI resources NR-54982), and hCoV-19/England/204820464/2020 (B.1.1.7, BEI Resources NR54000) were cultured in Vero.E6 cells for 3 days at 37°C and then the supernatant was clarified via centrifugation at 1,000*g* for 10 minutes. Virus stocks were stored at −80°C. The protocol was described in greater detail previously [22,39]. All work with authentic SARS-CoV-2 was performed in a biosafety level 3 (BSL-3) facility following institutional guidelines.

### In vivo mouse studies

All animal procedures were performed by adhering to the Institutional Animal Care and Use Committee (IACUC) guidelines of the Icahn School of Medicine at Mount Sinai IACUC and according to an approved protocol (IACUC-2014-0255). Research was conducted in concordance with the Animal Act PL99-158 (as amended). Six- to 8-week-old female, BALB/c mice were vaccinated via the intramuscular route with 3 μg of each respective protein with 1:1 mixture of AddaVax (Invivogen, San Diego, CA) in a total volume of 50 μL. After 3 weeks, mice were bled and vaccinated again. Three weeks later, mice were administered anesthesia via the intraperitoneal route and then intranasally transduced with AdV-hACE2 at 2.5 × 10^8^ plaque-forming units (PFUs) per mouse. Anesthesia was prepared using 0.15 mg/kg of body weight ketamine and 0.03 mg/kg xylazine in water. Five days later, all mice were infected with wild-type SARS-CoV-2 intranasally with 1 × 10^5^ PFU. Mice were humanely sacrificed on day 2 and day 5 for assessment of virus in the lungs. Lungs were homogenized using special tubes and a BeadBlaster 24 (Benchmark, Sayreville, NJ) homogenizer [40,41]. Viral load in the lung was quantified via a classic plaque assay [42].

### ELISA

Ninety-six-well plates (Immulon 4 HBX; Thermo Fisher Scientific, Waltham, MA) were coated with 2 μg/mL of each respective protein with 50 μL/well overnight at 4°C. The next morning, the coating solution was discarded, and each plate was blocked with 100 μL/well of 3% nonfat milk (AmericanBio; catalog no. AB10109-01000) in phosphate buffered saline containing 0.01% Tween (PBS-T). Blocking solution was kept on the plates for 1 hour at room temperature (RT). Serum samples were tested starting at a dilution of 1:50 with 1:5-fold subsequent serial dilutions. Serum samples were added to the plates for 2 hours at RT. Next, the plates were vigorously washed 3 times with 200 μL/well of PBS-T. Anti-mouse IgG-horseradish peroxidase (HRP)-conjugated antibody (Rockland; catalog no. 610–4302) was used at a dilution of 1:3,000 in 1% nonfat milk in PBS-T, and 100 μL of this solution was added to each well for 1 hour at RT. The plates were washed 3 times with 200 μL/well of PBS-T and dried on paper towels. Developing solution was prepared in sterile water (WFI; Gibco) using SigmaFast OPD (*o*-phenylenediamine dihydrochloride, catalog no. P9187; Sigma-Aldrich), and 100 μL was added to each well for a total of 10 minutes. To stop the reaction, 50 μL/well of 3 M hydrochloric acid was added, and the plates were read in a plate reader, Synergy 4 (BioTek, Winooski, VT), at an absorbance of 490 nanometers. Data were analyzed in GraphPad Prism 7.

### Neutralization assay

Twenty-thousand Vero.E6 cells were seeded per well in a 96-well cell culture plate (Corning; 3340) 1 day prior to performing the assay. Serum samples were heat inactivated at 56°C for 1 hour prior to use. Serum dilutions were prepared in 1× minimal essential medium (MEM; Gibco) supplemented with 1% FBS. Each virus was diluted to 10,000 50% tissue culture infectious doses (TCID_50_s)/mL, and 80 μL of virus and 80 μL of serum were incubated together for 1 hour at RT. After the incubation, 120 μL of virus–serum mixture was used to infect cells for 1 hour at 37°C. Next, the virus–serum mix was removed and 100 μL of each corresponding dilution was added to each well. A volume of 100 μL of 1X MEM were also added to the plates to get to a total volume of 200 μL in each well. The cells were incubated at 37°C for 3 days and then fixed with 10% paraformaldehyde (Polysciences, Warrington, PA) for 24 hours. The next day, cells were stained using a rabbit anti-nucleoprotein antibody (Invitrogen; PA5-81794) as primary antibody and a goat anti-rabbit secondary antibody conjugated to HRP (Invitrogen; 31460). This protocol was adapted from an earlier established protocol [22,35,43].

### Antigenic cartography

A target distance from a serum to each virus is derived by calculating the difference between the logarithm (log_2_) reciprocal neutralization titer for that particular virus and the log_2_ reciprocal maximum titer achieved by that serum (against any virus). Thus, the higher the reciprocal titer, the shorter the target distance. As the log_2_ of the reciprocal titer is used, a 2-fold change in titer will equate to a fixed change in target distance whatever the magnitude of the actual titers. Antigenic cartography [23] was then used to optimize the positions of the viruses and sera relative to each other on a map, minimizing the sum-squared error between map distance and target distance. Each virus is therefore positioned by multiple sera, and the sera themselves are also positioned only by their distances to the viruses. Hence, sera with different neutralization profiles to the virus panel are in separate locations on the map but contribute equally to positioning of the viruses. The antigenic cartography software used was written by Sam Wilks and is available as free and open-source software from https://www.antigenic-cartoraphy.org.

## Supporting information

S1 Fig Neutralization over binding ratio varies for each group. **(A)** The neutralization over binding ratio was calculated and depicted for each group against the homologous virus and homologous spike protein. Statistical analysis was performed with an ANOVA corrected for multiple comparisons, and the *p*-values are indicated when statistical significance was present. **(B-E)** Neutralization over binding ratios are shown for groups vaccinated with wild-type spike protein **(B)**, B.1.1.7 spike protein **(C)**, B.1.351 spike protein **(D)**, and P.1 spike protein **(E)**. Underlying raw data can be found in the S1 Data.
(TIF)

S1 Data This file contains all raw data underlying the figures. (XLSX)

## Abbreviations

AUC: area under the curve
BSL-3: biosafety level 3
COVID-19: Coronavirus Disease 2019
ELISA: enzyme-linked immunosorbent assay
FBS: fetal bovine serum
hACE2: human angiotensin converting enzyme 2
HRP: horseradish peroxidase
MEM: minimal essential medium
NTA: nitrilotriacetic acid
NTD: N-terminal domain
RBD: receptor binding domain
RT: room temperature
SARS-CoV-2: Severe Acute Respiratory Syndrome Coronavirus 2
TCID_50_s: 50% tissue culture infectious doses
VoC: variant of concern
