# Supplementary figures and images for: Vaccination with SARS-CoV-2 variants of concern protects mice from challenge with wild-type virus
Source: PLoS Biol. 2021 Dec 16;19(12):e3001384. doi: 10.1371/journal.pbio.3001384 (PMC8758087; doi:10.1371/journal.pbio.3001384)

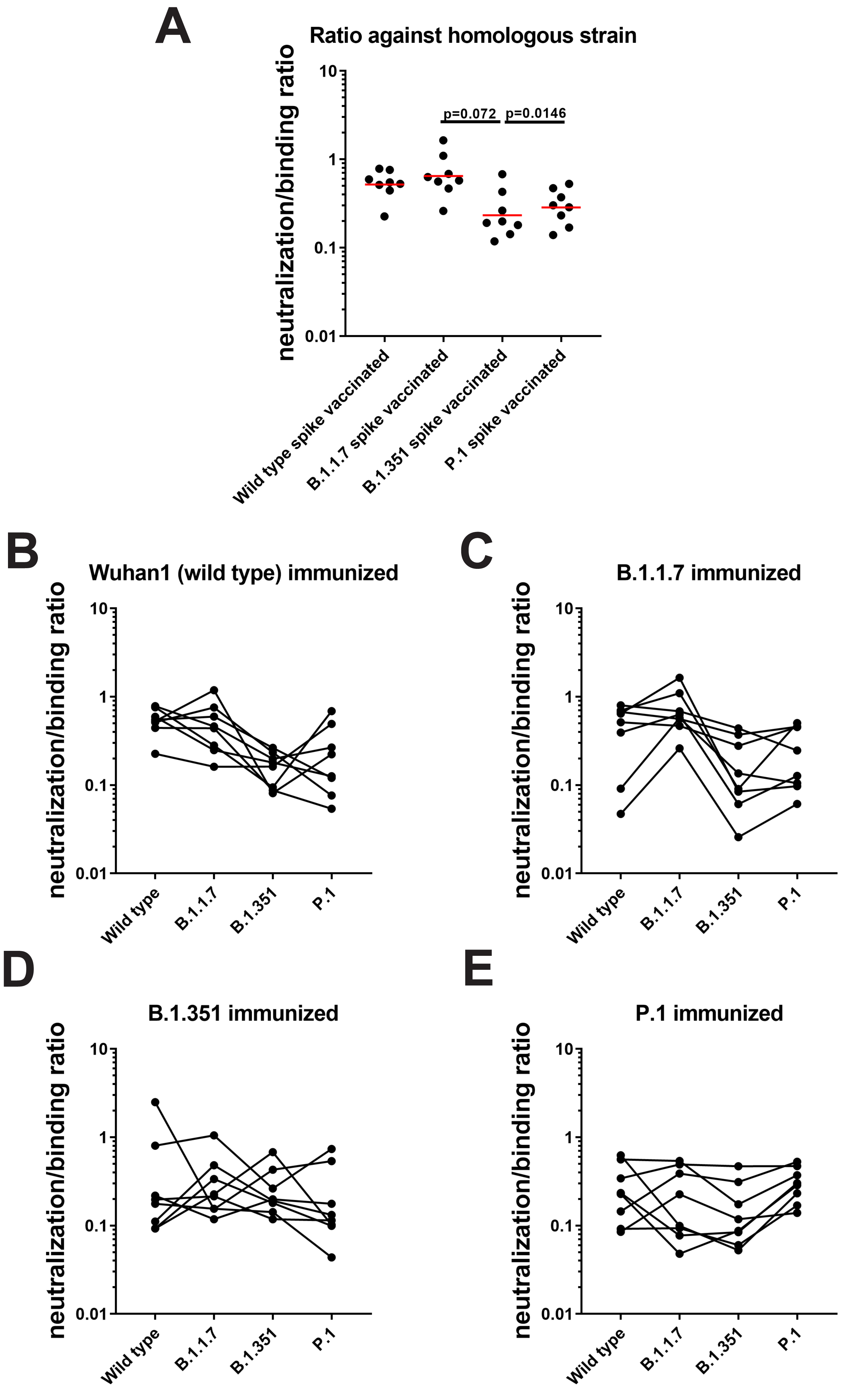

Supplement: S1 Fig — (A) The neutralization over binding ratio was calculated and depicted for each group against the homologous virus and homologous spike protein. Statistical analysis was performed with an ANOVA corrected for multiple comparisons, and the p-values are indicated when statistical significance was present. (B-E) Neutralization over binding ratios are shown for groups vaccinated with wild-type spike protein (B), B.1.1.7 spike protein (C), B.1.351 spike protein (D), and P.1 spike protein (E). Underlying raw data can be found in the S1 Data. (TIF) [file pbio.3001384.s001.tif]
